# Modeling Regulation of Zinc Uptake via ZIP Transporters in Yeast and Plant Roots

**DOI:** 10.1371/journal.pone.0037193

**Published:** 2012-06-08

**Authors:** Juliane Claus, Andrés Chavarría-Krauser

**Affiliations:** 1 Center for Modelling and Simulation in the Biosciences, Universität Heidelberg, Heidelberg, Germany; 2 Interdisciplinary Center for Scientific Computing, Universität Heidelberg, Heidelberg, Germany; Semmelweis University, Hungary

## Abstract

In yeast (*Saccharomyces cerevisiae*) and plant roots (*Arabidopsis thaliana*) zinc enters the cells via influx transporters of the ZIP family. Since zinc is both essential for cell function and toxic at high concentrations, tight regulation is essential for cell viability. We provide new insight into the underlying mechanisms, starting from a general model based on ordinary differential equations and adapting it to the specific cases of yeast and plant root cells. In yeast, zinc is transported by the transporters ZRT1 and ZRT2, which are both regulated by the zinc-responsive transcription factor ZAP1. Using biological data, parameters were estimated and analyzed, confirming the different affinities of ZRT1 and ZRT2 reported in the literature. Furthermore, our model suggests that the positive feedback in ZAP1 production has a stabilizing function at high influx rates. In plant roots, various ZIP transporters play a role in zinc uptake. Their regulation is largely unknown, but bZIP transcription factors are thought to be involved. We set up three putative models based on: an activator only, an activator with dimerization and an activator-inhibitor pair. These were fitted to measurements and analyzed. Simulations show that the activator-inhibitor model outperforms the other two in providing robust and stable homeostasis at reasonable parameter ranges.

## Introduction

Zinc is a heavy metal and micronutrient that plays an important role in all living organisms and is particularly essential for the growth of higher green plants [1]. It is part of the functional subunits or cofactor of more than 300 proteins, among them the class of zinc-finger-proteins as well as RNA-polymerases. In addition, it has been reported to protect plant cells from oxidative stress mediated by reactive oxygen species (ROS) [2] and may act as an intracellular second messenger [3].

In higher doses, however, zinc becomes toxic. Toxicity is far less frequent than deficiency, but likely in plants growing on contaminated soils, e.g. in mining or industrial areas. Most plants react to elevated zinc levels with toxicity syndromes, such as reduced growth and leaf chlorosis [4]. Only specialized zinc-hyperaccumulating species are able to tolerate high levels without impairment [5]. In order to do so, they possess mechanisms for both the increased uptake of zinc from the soil and its sequestration and detoxification [6]. These mechanisms are subject of ongoing research, as they implicate interesting applications in phytoremediation or nutritional enhancement [7].

Avoiding both deficiency and toxicity, plants need to take up their required amounts of zinc. Unlike animals they cannot adapt their nutrition accordingly, but depend on the zinc content of the soil. This content may vary considerably in different locations and under different conditions. How are plants able to adapt to this variety?

Charged zinc ions are unable to cross cell membranes freely [8]. Instead, they are taken up by specialized transporter proteins. To provide a sufficient zinc uptake without reaching toxicity, these transporters need to be tightly regulated. The regulatory mechanism has to consist of two parts: sensing of the intracellular zinc concentration and reaction to changes by controlling the amounts of zinc transporters. Sensing of changes in zinc concentrations must be very sensitive, because the actual available zinc concentration within the cells is believed to be very small. Zinc ions bind to various intracellular proteins, are chelated and sequestered into specific cellular compartments, such as the vacuole [9]. Therefore, although the total zinc content in the cells may be in a millimolar range, the actual concentration of free zinc ions is estimated to be much lower. Earlier investigations place it in a femtomolar range [10], while more recent results suggest nanomolar ranges [11], [12]. Zinc influx carriers are thought to be regulated by this pool of free zinc ions plus ions that are loosely bound to chelator proteins and can be set free to bind to other proteins with higher affinity. Our models will be based on this free and easily accessible zinc.

### Models of homeostasis

Homeostatic regulation in biological systems is based on genetic regulatory systems, and ultimately, on concentrations. These are positive, which constrains the possibilities of control substantially. In [13] the positiveness constraint of a robustly regulating enzyme was shown to lead to the need for two separate control mechanisms: for influx and efflux. The homeostatic model proposed in [13] is
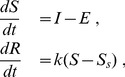
(1)where 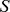 is the regulated species, 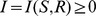 and 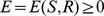 are the influx and efflux, respectively, 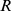 is the regulator, 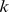 is a coefficient (not necessarily positive) and 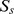 is the set point concentration. The above model may result in non-physical negative concentrations of the regulator [13]. Independently of the type of mechanism sought after, the negative term in 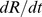 needs certain properties to achieve robustness based on positive concentrations. The approach is to have a term which is linear in 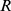 for small 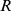 (positiveness), but becomes almost independent of 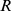 for larger 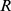 (robustness) [13].

Eq. (1) is an oversimplification of homeostatic control in cells, as substantially more complex mechanisms are needed [compare Eq. (3)]. Also the concept of perfect control is an idealization. Control of zinc fails in cells for low and high external concentrations. The presence of oscillations in perfect homeostasis, [14], poses a problem to living organisms. Strong oscillations could lead to transient, very high and potentially lethal concentrations. Prescinding from perfect regulation could be a compromise between avoiding strong bursts and achieving good control.

Based on biological information available, we will develop several putative models of influx homeostasis in plant root cells. In the first part, a general influx regulation model based on an ordinary differential equation system describing gene expression of transporters will be developed and non-dimensionalized. Using the general model, the biological model for yeast in [15] will be translated into a corresponding mathematical model. This model is simplified and fitted to transcript level data via a non-linear optimization method [16]. The mathematical properties of the steady state are analyzed and discussed. In the second part, the experiences won with the yeast model are used to pose three models for plant roots. The possibilities are manifold, for which reason we restrict the models to the most simple cases of: activator only, activator with dimerization and activator-inhibitor.

## Methods

### General model

The zinc homeostasis mechanisms presented in this manuscript can be arranged into a general model, which will be developed in this section. Zinc homeostasis can be split into two components: short and long term regulation. Short term regulation is fast but rough, while fine tuning is done by long term regulation. The time scale of short term regulation is less than two hours in plant roots [17]. Long term regulation has a substantially larger time scale of several hours, days, weeks, etc.

We are interested here in short term regulation, which is local in the sense that the processes occur at the level of single cells in plant roots. Other signals besides the fluxes seem not to be transmitted between cells or tissues. This is probably not the case for long term homeostatic control, which might rely on signals transmitted from tissue to tissue. Therefore, the short term response in plant roots and yeast cells is assumed to follow similar laws that can be subdivided into the phases
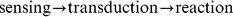
(2)The zinc status is measured in the sensing phase, decisions are taken in the transduction phase and changes in cytosolic concentration occur in the reaction phase. As mentioned in *Models of homeostasis*, both influx and efflux can be adapted to achieve homeostatic control. In plant roots as well as in yeast cells, adaptation of the expression of influx transporters poses the major component of zinc regulation [17], [18].

Based on the concept presented in Eq. (2), the models considered in this manuscript have the following structure
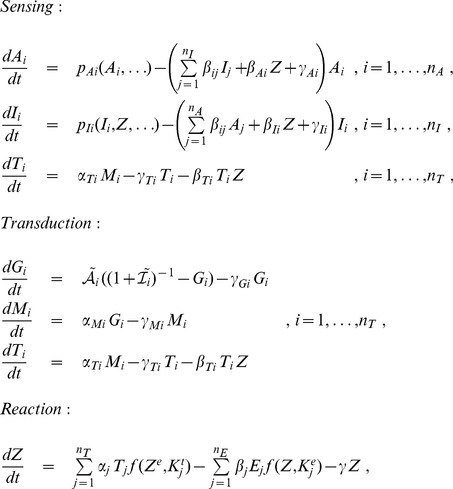
(3)where 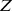 and 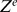 are the cytosolic and external zinc concentrations, respectively, 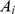 are activators, 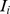 inhibitors, 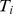 and 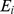 influx and efflux transporters, respectively, 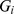 and 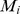 the levels of gene expression and mRNA of 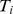, respectively, and 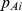 and 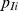 are model dependent production terms. The total activation and repression are
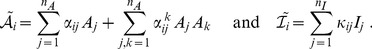
(4)The function 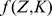 describes saturation of the transporters
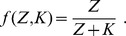

*Sensing* is assumed to take place at the level of the activators 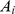 and inhibitors 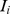. The possibility that the transporters 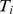 sense the cytosolic zinc concentration 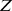 directly was also introduced. To achieve regulation, the total activation 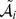 has to decrease with higher 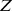 values (see *Models of Homeostasis*). *Transduction* is modeled in the usual way [19]. Three equations per protein are needed, namely for: gene activity 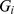, transcription into 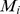 and translation into 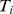. The activators are introduced as essential transcription factors, which activate the gene transcription. The quadratic form in Eq. (4) allows to include dimerization. The inhibitors inhibit either the activators or repress through 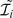 directly gene activity. Gene repression was assumed to be non-competitive and fast compared to activation, i.e. it is in quasi-equilibrium and 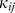 are equilibrium constants. *Reaction* is described by an equation for the cytosolic zinc concentration, which contains essentially the difference between influx and efflux mediated by 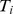 and 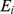, respectively, and a transporter independent consumption −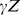. Regulation of the efflux transporters 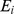 was left out of Eq. (3), as these vary only slightly in roots and no information on yeast was available. If included into the model, these proteins would follow a similar transduction system as the influx transporters 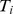.

Non-dimensionalization of *Transduction* in Eq. (3) is straightforward using
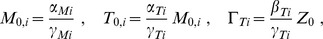
and the non-dimensionalized total activation and repression
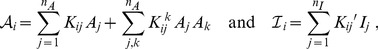
(5)with
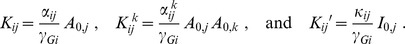

*Reaction* is non-dimensionalized by choosing
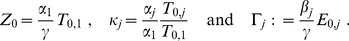
Non-dimensionalization of *Sensing* depends on the particular structure of the production terms. The decay terms can be non-dimensionalized choosing
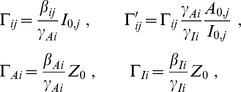
while the productions terms still have to be non-dimensionalized accordingly
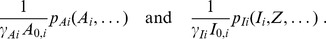



### Numerical Methods

The ordinary differential equation systems were simulated with either an explicit eighth-order Runge-Kutta method or an implicit Rosenbrock stepper for stiff differential equations. Steady states were calculated by Newton's method in combination with a path following method for varying parameters. Jacobians were calculated analytically. The model parameters were determined by fitting the model to measurements. For this purpose, Brent's algorithm was applied to minimize 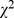 [16], [20]. The standard deviation of a measurement was assumed to be proportional to its value and the relative error (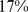) was chosen such to obtain a reduced 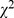 of the order of one. This way, low and high values had the same weights and were fitted equally well. Penalties were added to 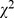 to avoid negative parameter values. The confidence intervals were obtained by calculation of the covariance matrix via the Hessian of 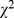 [20]. The measurements in [15], [21] were combined and scaled correctly. Scaling factors were in part included into the fitting process while others were prescribed with given values (personal communication of D. Eide).

## Results and Discussion

### Yeast

**Figure 1:**
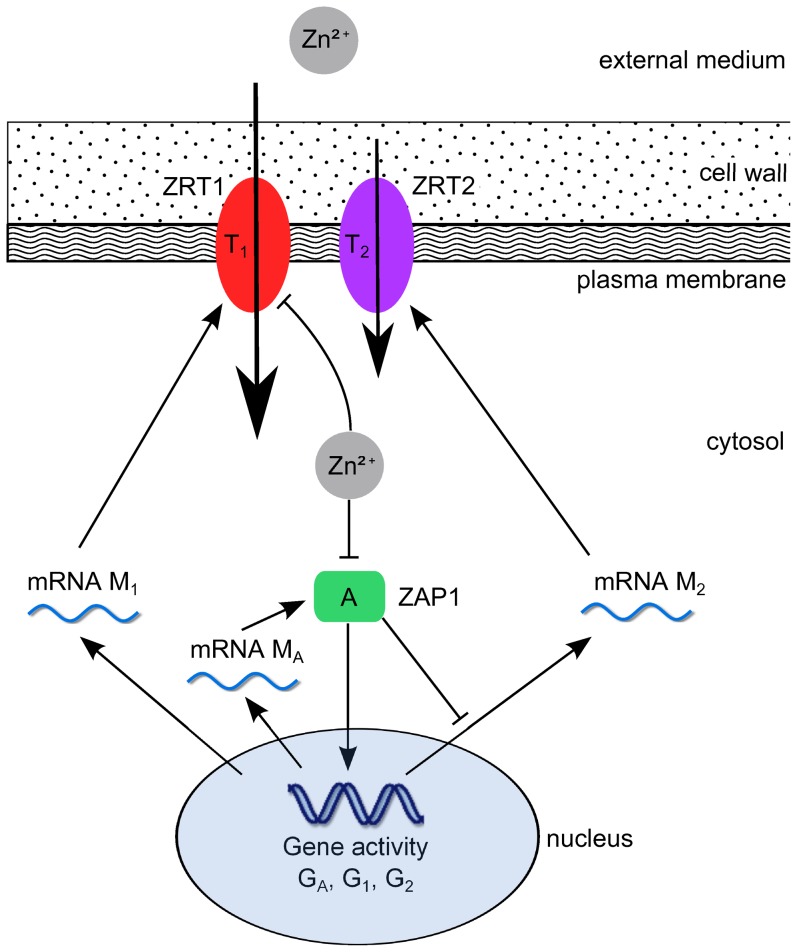
Yeast: scheme of zinc influx regulation model. ZAP1 is inactivated by zinc and activates transcription of the transporters ZRT1 and ZRT2.

The regulation of zinc uptake in yeast cells (*Saccharomyces cerevisiae*) has been studied in much detail and found to be a combination of two systems with high and low affinity for zinc ions. A similar distribution of high and low affinity transporters has also been found in wheat [22] and is thought to exist in other plants as well [23]. A schematic overview of the system can be seen in Fig. 1. Zinc ions are transported with high affinity by ZRT1 (zinc-responsive transporter) and with low affinity by ZRT2, which both belong to the ZIP (zinc-, iron-permease) family. ZRT1 has been found to be strongly regulated by the intracellular zinc concentration and almost exclusively active under conditions of zinc deficiency [24]. ZRT2 has been reported to guarantee a basic zinc uptake level under normal zinc-replete conditions [25] while being repressed under zinc deficiency [21]. Further studies have shown that both *ZRT1* and *ZRT2* are activated by the transcription factor ZAP1 (zinc-dependent activator protein) [15], which binds to so-called zinc responsive elements (ZREs) in the promoter regions of the respective genes. Under conditions of elevated zinc concentrations, the activity of ZAP1 is reduced and production of ZRT1 and ZRT2 decreases. Inactivation of ZAP1 occurs most likely by direct binding of free zinc ions, although further signaling molecules may also be involved in this process. By binding to its own promoter region, ZAP1 regulates its transcription introducing a positive feedback mechanism and presumably allowing an even stronger response to zinc-limiting conditions [18]. In addition to the transcriptional regulation, ZRT1 is also regulated by a post-translational mechanism [18]. While it is a stable membrane protein under zinc deficient conditions, ZRT1 is ubiquinated and subjected to endocytosis for high intracellular zinc levels. The details of this mechanism have been investigated in [26], but it is yet unknown whether zinc ions bind directly to ZRT1 to induce its ubiquitination, or whether other zinc-binding proteins are involved. It has been proposed that the combination of transcriptional and post-translational regulation allows for a very quick response to changing environmental conditions and thus prevents a toxic zinc shock [18].

#### Model

As described above, zinc uptake regulation in yeast comprises the two zinc transporters ZRT1 and ZRT2, as well as the transcription factor ZAP1 as the only activator, which is directly inhibited by zinc ions without an inhibitor. The production of the activator, which corresponds to the term 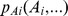 in the general model Eq. (3), is a system of *Sensing*, *Transduction* and *Regulation* by itself, because ZAP1 acts as its own transcription factor through a positive feedback loop. While *ZRT1* is simply activated by ZAP1, *ZRT2* is both activated and repressed by the same molecule [21]. Therefore, we assume a model with two binding sites of ZAP1 close to the *ZRT2* gene, one activating and one repressing. The total inactivation 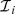 [see Eq. (5)] introduces this mechanism into the general model Eq. (3). Here, the inhibitor is equal to the activator and only the *ZRT2* gene is affected: 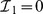 and 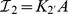.

Following the framework of the general model and the non-dimensionalization derived in *General Model*, we obtain the following system:
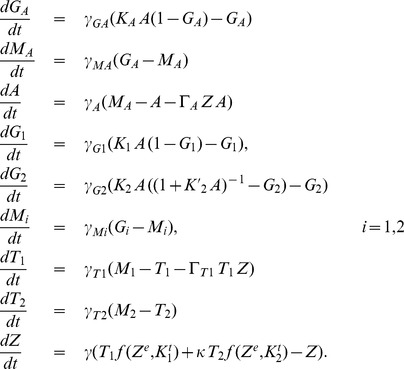
(6)The post-translational regulation of ZRT1 is given by the term 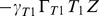. For simplicity the term 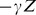 accounts for all zinc consumption processes. These may include export from the cell through zinc efflux transporters, sequestration into the vacuole and other compartments as well as irreversible binding and chelation of zinc by various proteins in the cytoplasm.

The trivial solution (all species zero) is a steady state of Eq. (6). There is at least one non-trivial steady state, which for the activator ZAP1 can be written as a function of the intracellular zinc concentration
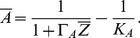
(7)For 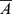 not to become negative, this equation poses the condition 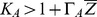, which implies that for large 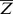 the non-trivial and trivial solutions cross. A detailed analysis of this case is presented below. The case of total deficiency (i.e. 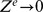) brings insight into some of the parameters. As expected, we find 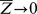, which means that 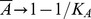. From the biological point of view, 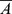 is expected to shoot to a value close to 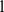 for total deficiency, which implies 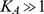. Assuming that 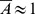 for 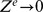, the concentrations of the transporters 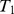 and 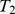 behave for 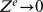 as
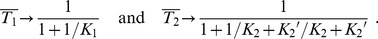
High affinity of ZRT1 and low affinity of ZRT2, i.e. 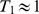 and 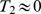 for 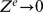, are obtained when the conditions 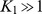 and 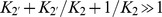 are fulfilled. Considering 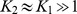, the second condition is essentially 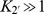. Expression of *ZRT2* is maximal for a ZAP1 concentration of 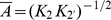, while expression of *ZRT1* rises monotonically with 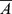 and approaches its highest value for 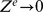. For a given activation 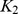, repression 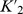 has to be large to shift the expression maximum towards low 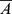 and high 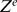.

**Figure 2:**
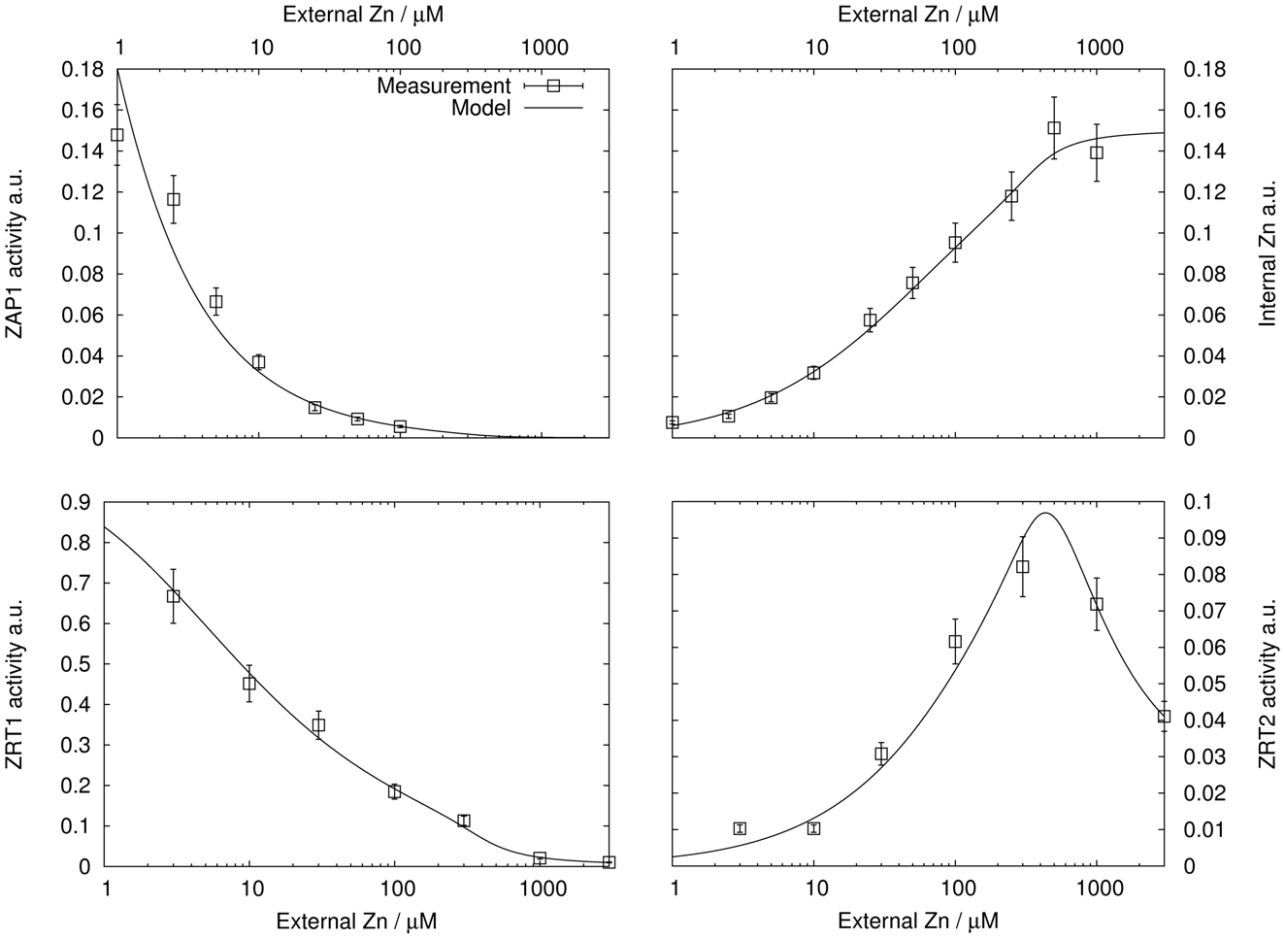
Yeast simulations. Comparison between measurements and simulated steady states of ZAP1, internal zinc, ZRT1 and ZRT2 for varying external zinc concentration. Measurements: ZRT1 and ZRT2 from [21], ZAP1 and zinc from [15].

Using the quantitative data measured in [15] and [21], we estimated the model parameters by a least-square method. These measurements are stationary, and thus, the system reduces to one with the four unknowns 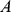, 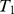, 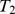 and 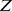. The parameters obtained are listed in Table 1. These clearly reflect the above conditions for 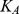, 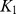, 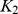 and 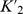. The model reproduces very well the measurements (Fig. 2). Our model suggests that in the steady state, vacuolar storage affects homeostasis only via a contribution to a simple linear consumption term (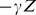). Regulation of vacuolar storage seems not to be important to explain the data in Fig. 2. However, it might be important for dynamics and buffering of short-time zinc excess. To be able to model this kind of situations properly, experimental data on the partition of zinc into the vacuole and cytosol would be needed.

**Table 1:** Yeast: parameters.

Parameter	Value	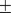 s.d.
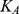	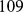	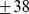
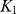	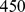	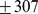
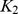	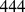	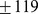
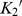	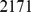	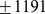
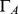	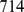	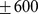
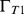	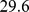	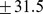
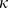	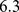	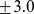
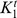 / 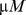	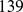	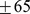
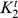 / 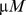	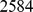	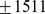

Parameters values and standard deviations obtained by fitting the model to measurements published in [15] and [21].

#### Roles of ZRT1 and ZRT2

In [15], ZRT1 and ZRT2 were proposed to play different roles in zinc uptake of yeast cells. While ZRT1 is most active only in zinc-deficient cells, ZRT2 is transiently active also in zinc-replete cells with external zinc concentration around 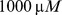. This implies that under low external zinc concentrations ZRT1 dominates the overall zinc uptake, while under high external zinc concentration, ZRT2 acts as the major transporter. This behavior is confirmed by our model.

**Figure 3:**
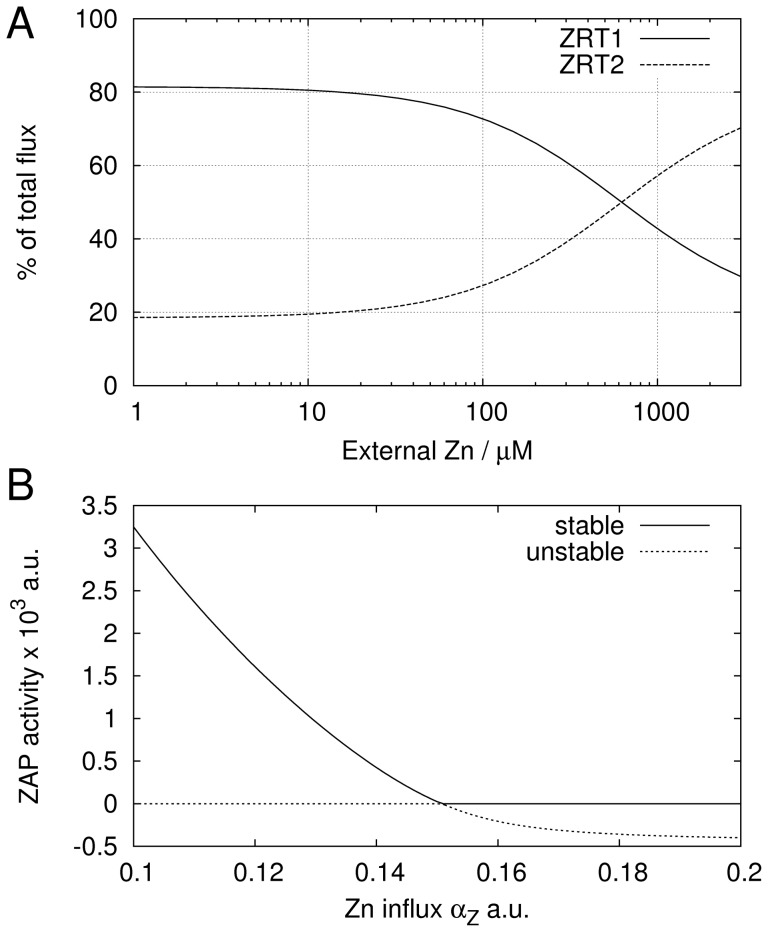
Yeast: Role of ZRT1 and ZRT2 and ZAP1 feedback. A, contributions of ZRT1 or ZRT2 to the total zinc influx for varying external zinc concentration. B, ZAP1 activity for varying values of ZRT independent influx 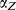. The stable solution is marked with a solid line, the unstable solution is dotted.

Fig. 3A presents the relative contributions to the total flux. At low external concentrations ZRT1 is responsible for about 80% of the influx, while at replete conditions (above 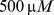) 70% of the influx can be attributed to ZRT2. ZRT1 seems indeed to act as a high affinity transporter with a Michaelis constant 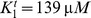, while ZRT2 has less affinity reflected by a substantially larger 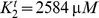. A similar ratio was found in [25], although their values are several orders of magnitude lower. This discrepancy stems from the assumption made in [25] that the response is based on saturation of a constant number of transporters (pure Michaelis-Menten kinetics without regulation). However, regulation might have taken place during the time of measurement, thereby influencing the number of transporters and thus the uptake rate. Fig. 6A in [254] supports this hypothesis, as the uptake rate is maximal at ca. 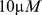 and a maximum cannot be explained with Michaelis-Menten. The low published values are reproduced with our model by fitting Michaelis-Menten to the simulated uptake rates (including regulation effects). The approach presented here delivers the Michaelis-Menten constants of the sole proteins und should correspond to values of purified proteins measured in vitro. Higher 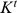 values than the ones published in [25] seem also more plausible, because otherwise the transporters would be saturated already at moderate external zinc concentrations. The affinity of the ZRT1 and ZRT2 systems are not completely determined by 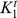 and 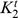, respectively. These constants have to be larger than the optimal concentration of the corresponding system, as saturated transporters cannot pass information on external zinc status (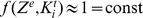 for 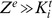). The optimal concentration for *ZRT1* is at total deficiency, while *ZRT2* is most active at 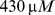 (Fig. 2).

A strong repression of *ZRT2* is essential to achieve a maximal expression at high external zinc concentrations (see Table 1). However, a strong repression also results in lower gene activities, which explains the low expression level of *ZRT2* compared to *ZRT1* (Fig. 2 and [21]). To compensate the lower expression level, ZRT2 needs to transport zinc at higher rates or more copies need to be produced. This is reflected by the coefficient 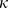, which suggests that ZRT2 is six times more effective in transporting zinc than ZRT1. Assuming that the ZRT1 and ZRT2 molecules transport zinc at a similar rate, 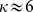 could indicate posttranslational regulation of ZRT1. Direct posttranslational regulation via 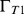, however, was shown not to be significant here (F-test: 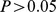). The higher transport efficiency of ZRT2 explains also why at low zinc concentrations, e.g. at 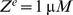, ZRT2 contributes about 20% to the total flux although its expression level is substantially lower.

*ZRT1* and *ZRT2* were found to be activated equally well by ZAP1, as reflected by the insignificantly small difference between 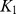 and 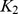. The self-activation constant 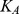 of *ZAP1* is four times smaller than 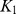 and 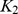. This suggests that *ZRT1* and *ZRT2* have more ZAP1-binding promoters than *ZAP1*, which is in concord with experimental results [15].

#### ZAP1 transcriptional feedback

The feedback loop generated by ZAP1 acting as its own transcription factor introduces interesting properties into the model. In [18] this feedback was proposed to allow a stronger reaction to zinc-limiting conditions. In contrast, our model suggests that the advantage is rather for zinc-replete conditions. The steady state Eq. (7) of ZAP1 becomes negative for 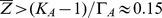 and crosses the trivial steady state. Unless these two steady states exchange their roles, the model would become non-biological at the bifurcation. Based on the fitted parameters, the bifurcation is normally reached at very high external zinc concentrations. To examine the behaviour of the model at the bifurcation, we introduced a ZRT1- and ZRT2-independent path into the cell. Such a path could for example be another transporter not regulated by ZAP1 and shifts the bifurcation towards lower 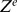. Without considering any details of these processes, the simplest modification is to include an additional constant zinc influx term 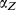 to the last line in Eq. (6). The bifurcation is illustrated in Fig. 3B. There are at least two steady states, where one is trivial (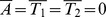 and 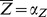) and one is positive for small 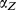 (other negative steady steady states exist). The stability of these are exchanged at the bifurcation. For low 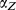 the positive steady state is stable, while the trivial steady state is unstable. After the steady states cross at the bifurcation, the trivial solution becomes stable while the now negative steady state becomes unstable. The positive steady state is literally trapped by the trivial steady state. From the biological view the ZAP1 feedback allows the system to completely switch off the expression of *ZAP1* and thus of *ZRT1* and *ZRT2*. In a mechanism without feedback, *ZAP1* expression would just decrease asymptotically towards zero for increasing zinc influx. Therefore, we conclude that the feedback of ZAP1 is advantageous for zinc-replete conditions.

### Plant roots

In plants, zinc is taken up from the soil and transported into the root cells. Unlike in yeast, zinc needs to be transported into further tissues: xylem, stem, leaves, etc. Numerous proteins are involved in the transport, which can be grouped into three families: ZIP, HMA (heavy-metal-ATPases) and MTP (metal tolerance protein), also known as CDF (cation diffusion facilitator). Members of the ZIP family are believed to act as influx carriers, including uptake from the soil (similar to ZRTs in yeast). HMAs accomplish efflux of zinc, e.g. from roots into xylem vessels, while MTPs are involved in sequestration into compartments, such as the vacuole [27]. The main influx transporters of root cells are ZIP1, ZIP2, ZIP3, ZIP9, and IRT3 (iron-responsive transporter) [28], while ZIP4 localizes to the chloroplast [23]. These transporters are highly expressed under conditions of zinc deficiency, whereas their expression decreases quickly when zinc is added to the media [17]. The exact mechanism of this regulation is still unknown. Recent results have shown that at least *ZIP4* in *Arabidopsis thaliana* is regulated by transcription factors of the basic-region leucine zipper (bZIP) family: bZIP19 and bZIP23 [17]. These factors bind to a ZDRE (zinc deficiency response element), which has been found not only in the upstream region of *ZIP4*, but also of *ZIP1*, *ZIP3*, *ZIP9* and *IRT3*. Therefore, it is reasonable to assume similar regulation for these ZIP transporters.

Unlike the ZAP1 transcription factor in yeast (see Section *Yeast*), bZIP19 and bZIP23 transcription factors do not have a zinc binding site [29]. It is unclear how they sense the intracellular zinc status. Existence of further players that bind zinc and act as inhibitors of bZIP19 and bZIP23 have been proposed [30]. Transcription factors of the bZIP family have been studied in other regulatory networks and are known to be regulated post-transcriptionally in various ways [31]. Generally, bZIP transcription factors (in particular bZIP19 and bZIP23) are known to dimerize [32]. They are partially redundant [29] and it is believed that they preferentially form homodimers, but may also constitute heterodimers [33].

Our model focuses on the uptake of zinc into the root cell space without consideration of further transport. By restricting the model to this specific situation, a similar approach as the one for yeast can be applied. We start with a simple model based on only one zinc dependent activator. Hereafter, the advantage of dimerization is analyzed, and a third more involved model based on an activator-inhibitor pair is presented. Using the data in [17], some of the parameters are obtained via optimization. An F-Test is used to compare the models and select the most reasonable one. Finally, we analyze the relation between stability and robustness of the activator-inhibitor model.

#### Activator

**Figure 4:**
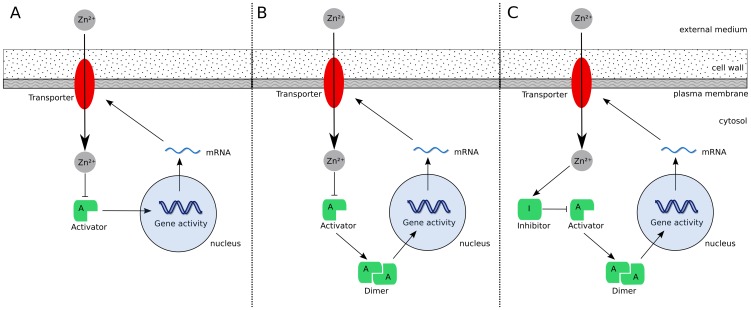
Plant roots: Scheme of the three models of zinc uptake regulation. A, Activator only [Eq. (9)], B, Activator with dimerization [Eq. (15)], C, Activator-Inhibitor model [Eq. (16)].

Here, we assume that regulation takes place by one zinc dependent transcription factor (see Fig. 4A for a scheme). In terms of the general model Eq. (3) we set 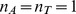 and 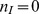 and avoid unnecessary notation by dropping indexes (e.g. 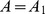 and 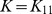, etc.). Sensing is assumed to take place only at the activator level (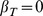). The possibility that the activator dimerizes is also ruled out (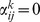). Efflux transporters are assumed to be non-saturable, which allows combining efflux/consumption into one term 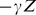. In contrast to the case of yeast, there is no specific information on the production of the activator available. To keep the system simple, we introduce a constant pool 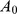 of activator, which is split into active, 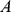, and inactive molecules, 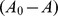. The net production is set to 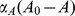 and
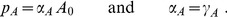
(8)The non-dimensionalized system is
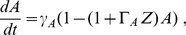


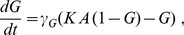


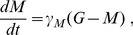
(9)

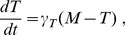


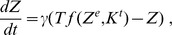
with two steady states
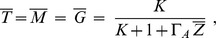


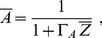
(10)

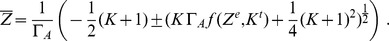
The steady state with 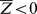 is biologically irrelevant and therefore not considered. For total deficiency, i.e. 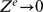, we find
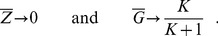
(11)Biology suggests that gene expression will shoot to a very high value, so 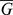 should be close to one. This implies: 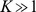. For replete conditions, i.e. 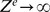,
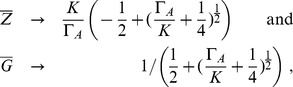
(12)where 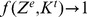 and 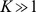 were used. Biology suggests that gene expression should be small for high external zinc concentrations, which implies
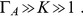
(13)For a given 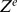, the steady state depends on three more parameters: 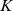, 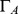 and 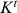. While 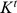 is a property of the transporters, 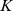 and 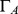 determine gene activity for extreme conditions. For ZIP1, a value 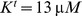 was published in [28] and used here. Assuming gene activity to reach at least 95% for total zinc deficiency, we obtain
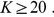
(14)Determination of 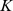 from measurements would need data at very low zinc concentrations, which is uncertain and was not available to the authors. For this reason, an empirical value of 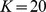 was used. The remaining parameter 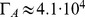 was obtained by fitting the model to published values of *ZIP3* expression [17]. All parameters are listed in Table 2.

**Table 2:** Plant roots: parameters.

Parameter	Act. only	Act. dimer.	Act./Inhib. dimer.
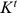 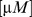 *	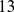	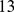	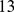
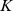	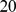	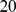	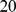
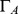	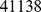	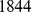	–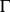	–	–	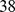
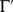	–	–	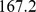
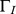	–	–	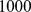
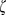 †	–	–	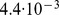
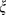 ‡	–	–	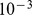

* Value for ZIP1, [28];

†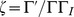;

‡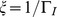.

Plant roots: parameters used in the simulation of the *activator only*, *dimerized activator* and the *dimerized activator-inhibitor* models.

**Figure 5:**
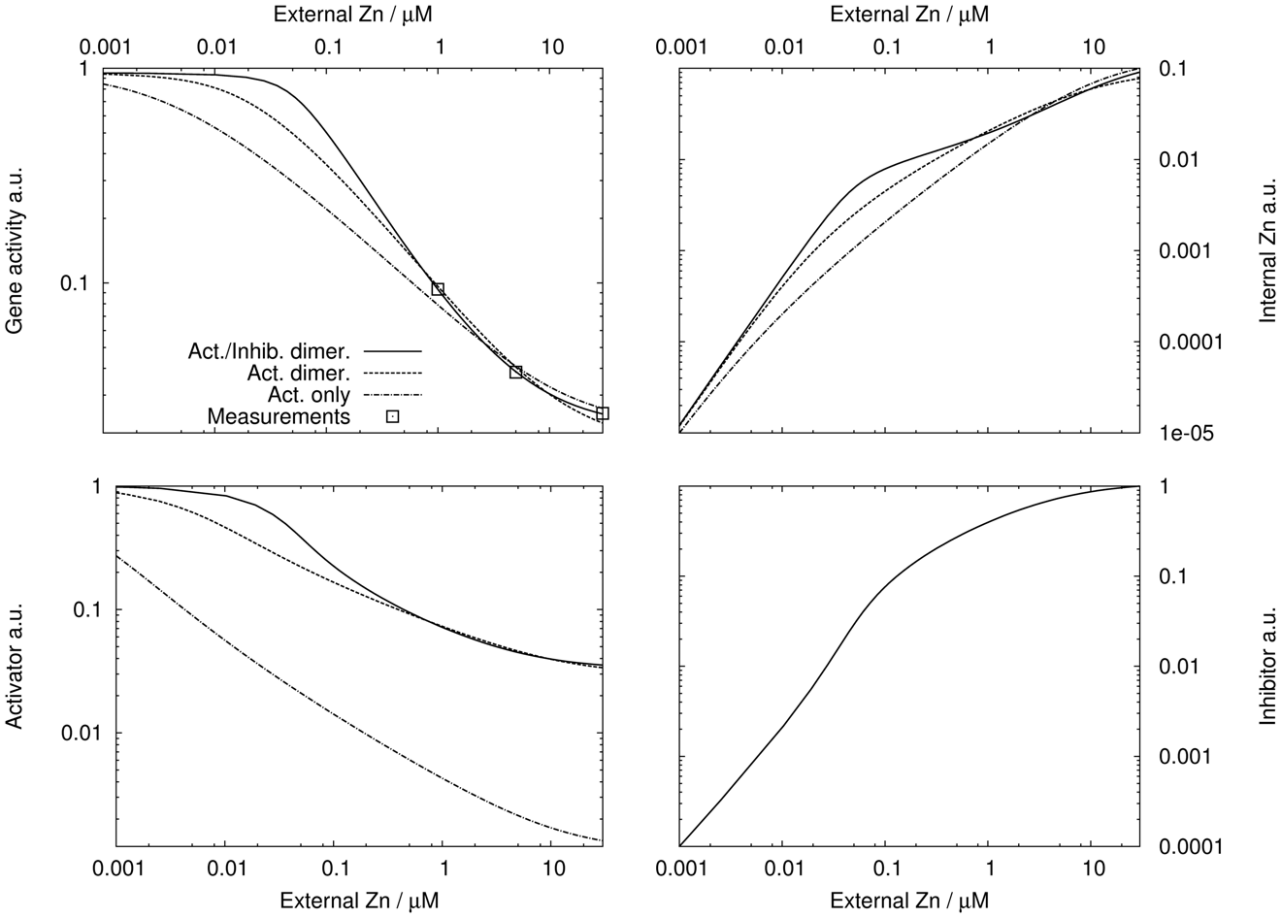
Plant roots: Steady states of the different regulation models. The model are: activator only (dash-dotted), dimerizing activator (dashed) and activator-inhibitor pair with dimerization (solid). Measurements of [17] are also shown.

The dash-dotted line in Fig. 5 shows the steady state of this model as a function of 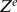. Gene activity decreases slightly for increasing 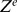 resulting in a continuously increasing internal zinc concentration. Regulation fails for extreme zinc conditions, i.e. undersupply at low 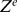 and oversupply for large 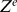. The reason for oversupply is the activator reacting insufficiently to changes in 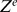. By adjusting 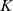 and 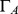, the model only offers the possibility to fix the maximum and minimum of gene expressions, but not the transition steepness between these. 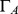 is also very large compared to the value determined for yeast (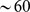 times larger; Table 1), rendering this simple activator-only model even more unlikely.

#### Dimerization

The transcription factors bZIP19 and bZIP23 are known to form dimers [32]. Assuming that only these dimers activate the gene yields 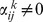 and 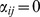 in the general mode. A scheme of the model is presented in Fig. 4B. The total activation is here 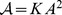, while the rest stays the same as in Eqs. (9) and (10), meaning that only gene activity needs to be adapted:
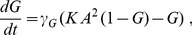
(15)

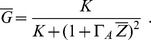
Gene activity reacts more sensitive to changes of zinc status than in the non-dimerizing case (Fig. 5, dashed line). The transition between gene on and off is steeper, rendering a more robust mechanism. Fitting the model to the measurements delivers 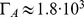, which is approximately 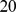 times smaller than in the non-dimerizing case and substantially closer to the value for yeast (Table 1). From an evolutionary point of view, dimerization allowed to down-regulate the transporters more strongly with less binding affinity. Also, by assuming that the standard deviations of the measured values are proportional to these, one finds that 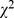 for the model with dimerization is less than half that of the one without when fitted to the measurements in [17]. In total, the model with dimerization statistically and qualitatively outperforms the above activator model, although both models have the same number of degrees of freedom. Aside from the statistical point of view, we find the dimer model more likely, because it results in more reasonable parameter values, comparable to those obtained in yeast. There is little data available for plants and future measurements spanning over a larger concentration range are needed to confirm this hypothesis.

#### Activator-Inhibitor

**Figure 6:**
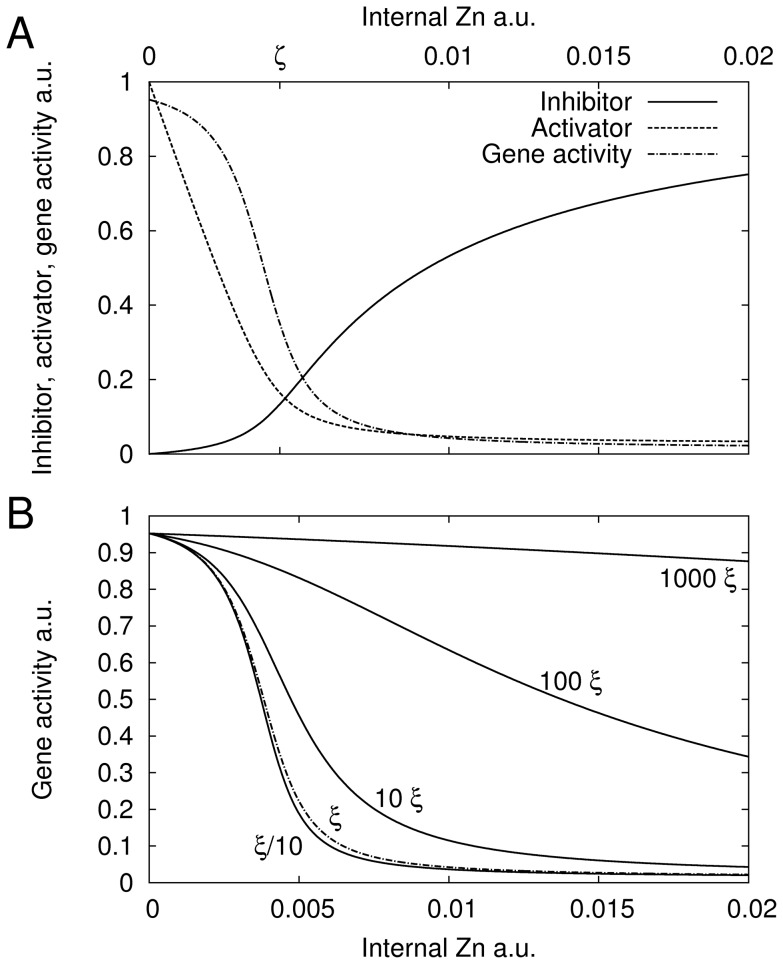
Plant roots: Activator-Inhibitor model with dimerization. A, steady state values of inhibitor (solid), activator (dashed) and gene activity (dash-dotted) in dependence of internal zinc concentration. B, steady state gene activity in dependence of internal zinc status for varying 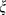. Dashed curve corresponds to the nominal 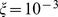.

Including dimerization delivered a better fit to the measurements than the activator only model. However, a systematic deviation for high values of 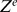 was found (Fig. 5, dashed line). Following the proposition in [30] of intermediate steps in sensing, we propose a mechanism involving an activator-inhibitor pair. Assume that these can interact while they are not bound to the DNA, that the pairs cannot activate the gene, and that zinc is sensed only by the inhibitor (Fig. 4C). Applying these assumptions to the general model Eq. (3) gives 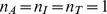. Dimerization again is included by using the total activation 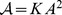. Production of activator is set as in the activator only model [Eq. (8)]. Sensing occurs at the level of the inhibitor:
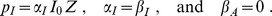
Transcription and translation are the same as in the dimerizing activator case. The equation for 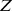 stays the same, meaning that the key differences to Eq. (9) are
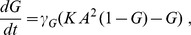


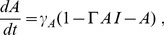
(16)

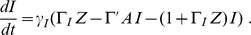
If 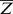 is considered to be a parameter in the above system, the steady state is
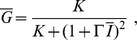


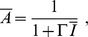


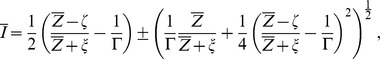
where 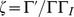 and 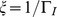. The solution with 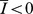 is biologically irrelevant. For totally deficient conditions, i.e. 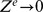,
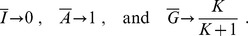
The case of very high external zinc concentration needs to include the expression for 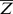. Instead of determining what happens for 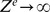, we determine the behavior for large internal concentrations, i.e. 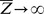:
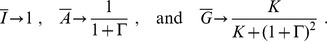
The same biological conditions as those listed in Eqs. (13) and (14) are found here. In contrast to the activator models, gene activity does not go to zero for 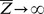. Again, the constants 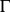 and 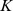 determine gene activity for extreme zinc levels. The steady state values depend on two more constants: 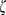 and 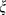. The meaning of these constants is found by the following reflection. The first term in 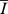 is zero for 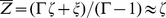. Is 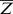 smaller than this value, the term is negative and has to be compensated by the slightly larger positive square root term, i.e. the inhibitor level 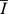 stays close to zero. Is 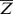 larger than this value, both terms are positive and the inhibitor level 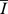 increases fast with 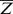. The activator is inhibited substantially and a strong reduction of gene activity is the consequence (compare Fig. 6A). Thus, 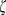 determines the internal zinc concentration for switching the gene from on to off. The constant 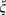 determines the steepness of the transition between the on and off states (Fig. 6B). A small 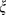 corresponds to a strong binding affinity 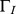 between zinc and inhibitor. The switching steepness is also affected by 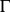, as it weights the first term under the root. Large 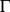 result in steeper switches with a similar effect as decreasing 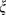 (Fig. 6B).

The activator-inhibitor model renders a better and more robust homeostatic control mechanism than the activator only models (Fig. 5). None of the proposed models shows the kind of perfect homeostatic behavior achieved using Eq. (1) with a zero order 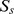. By simplifying the activator only models (with and without dimerisation) a system similar to Eq. (1) can be obtained, where 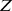 corresponds to the species and 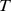 to the regulator. Compared to that model, the set point depends here on the external zinc concentration and explains why these models do not show much robustness (Fig. 5). The activator-inhibitor model, however, reacts similar to Eq. (1) within a small region around 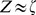, i.e. 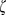 corresponds to 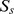 in Eq. (1). The reason is the steep genetic switch obtained by the inclusion of an inhibitor, which reacts strongly to the internal zinc status (Fig. 6A). Near the set point, gene activity and thus transporter concentration can vary substantially without affecting much the internal concentration. Fitting the model to the measurements delivered 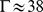 and 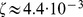 (Table 2). 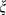 cannot be determined by a fit, because a robust mechanism is sought after and in that regime the model becomes almost independent of 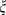 (compare Fig. 6B). Therefore, a value of the same order of magnitude as 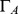 for yeast was used (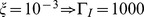 while 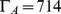 for yeast). The model describes the measurements very well (Fig. 5, solid line), which is also a consequence of the small number of degrees of freedom. No systematic deviation for large 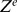 was found for this model. An F-Test showed that the activator-inhibitor is statistically more likely, even considering that it contains one more parameter (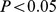).

#### Robustness and instability

In [14] perfect homeostatic control was shown to lead to undamped oscillations. In the case of a toxic substance, oscillations may cause lethal peaks. In view of this, the stability of the activator-inhibitor model was analyzed. Dynamics and stability depend on the time scales involved in the mechanism. The authors could not find suitable data for these. Similar values to those listed in [34] were used, where the products were assumed to decay four times slower than gene activity. The reader should keep in mind that the specific choice of the time scales influences stability, but the relation between robustness and instability found below should remain valid.

**Figure 7:**
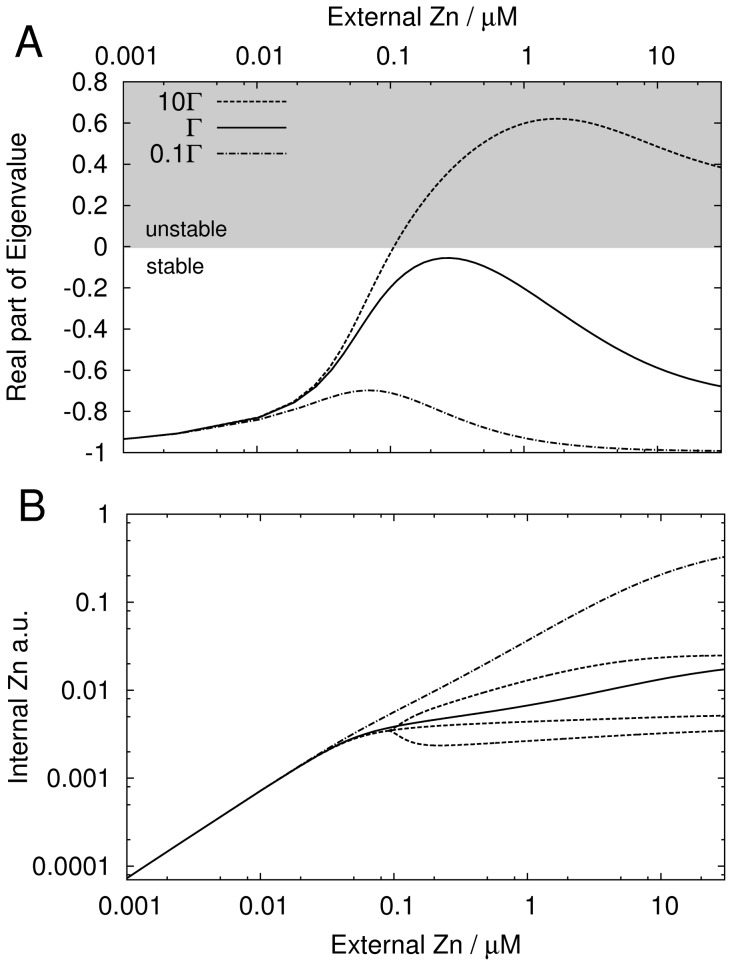
Plant roots: Robustness and stability. Robustness and stability of the activator-inhibitor model for 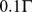 (dash-dotted), 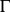 (solid) and 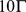 (dashed) and varying external zinc concentration. A, real part of largest eigenvalue. B, internal zinc concentration. Minimal and maximal values of limit cycle shown for unstable steady state (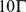).

Regarding robustness, a duality between the static and dynamic properties of the activator-inhibitor mechanism was found. Large 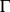 resulted in a steeper genetic switch and consequently the steady state internal zinc concentration varied less with 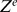 (Fig. 7B). At a first glance robustness of the mechanism seemed to increase with 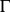. However, large 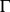 lead also to instability of the steady state and to undamped oscillations (Fig. 7A). Therefore, from a point of view of the dynamics, robustness decreased for increasing 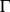. During one oscillation period, the internal zinc concentration reached up to 3.5 times the steady state value (oscillation amplitudes for 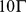 also shown in Fig. 7B), meaning that strong and possibly toxic periodic peaks of zinc were produced. These peaks exceeded the steady state concentration for the nominal 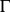. We conclude that toxicity for high external zinc concentrations could either occur because of stable high internal zinc concentrations (small 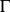) or due to toxic high amplitude oscillations (large 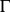). Reducing the perfectness of the homeostatic control could be a strategy to avoid strong zinc bursts, but cells might also use other mechanisms to damp strong oscillations, e.g. buffering and sequestration.
